# Predominantly diastolic coronary narrowing caused by iatrogenic left main dissection: deferral of stenting by combined angiographic- and ultrasound-based functional assessment

**DOI:** 10.1007/s10554-026-03706-9

**Published:** 2026-04-17

**Authors:** András Ágoston, Ádám Piricsi, Zsuzsanna Tokár, Michael Kest, Csaba András Dézsi, Zsolt Kőszegi

**Affiliations:** 1 Szabolcs–Szatmár–Bereg County Hospitals and University Teaching Hospital, Nyíregyháza, Hungary; 2 Kálmán Laki Doctoral School of Biomedical and Clinical Sciences, University of Debrecen, Debrecen, Hungary; 3 Faculty of Health and Sport Sciences, Department of Clinical Health Sciences and Nursing Győr, Széchenyi University, Győr, Hungary; 4 Department of Cardiology, Faculty of Medicine, University of Debrecen, Debrecen, Hungary

**Keywords:** Left main coronary artery, Iatrogenic coronary dissection, QFR, UFR, Murray law-based QFR

## Introduction

Iatrogenic dissection of the left main coronary (LM) artery is an uncommon but potentially life-threatening complication of coronary procedures. Immediate recognition and appropriate management are essential due to the large myocardial territory at risk [1]. Dynamic coronary stenoses are typically associated with systolic compression, such as in myocardial bridging [2, 3]. However, the occurrence of diastolic-dominant luminal narrowing has not been previously described.

We report a unique case of iatrogenic LM dissection extending into the LAD, characterized by predominantly diastolic coronary narrowing. The case highlights the value of multimodality imaging, including intravascular ultrasound (IVUS) and angiography-derived physiological indices, in guiding management decisions.

## Case Presentation

A 65-year-old woman with a history of arterial hypertension and type 2 diabetes mellitus presented with acute chest pain and dyspnea. Electrocardiography showed only mild ST–T abnormalities, while high-sensitivity cardiac troponin I was elevated (310 ng/L) with a rising trend to 6530 ng/L. Transthoracic echocardiography revealed reduced left ventricular ejection fraction with hypokinesis of the lateral and apical segments.

The patient was diagnosed with non-ST-segment elevation myocardial infarction and underwent coronary angiography. The right coronary artery was free of significant stenosis. The left main coronary artery appeared angiographically normal, with a mild proximal LAD lesion. A critical stenosis was identified in the obtuse marginal branch of the left circumflex artery and treated successfully with stent implantation.

**Fig. 1:**
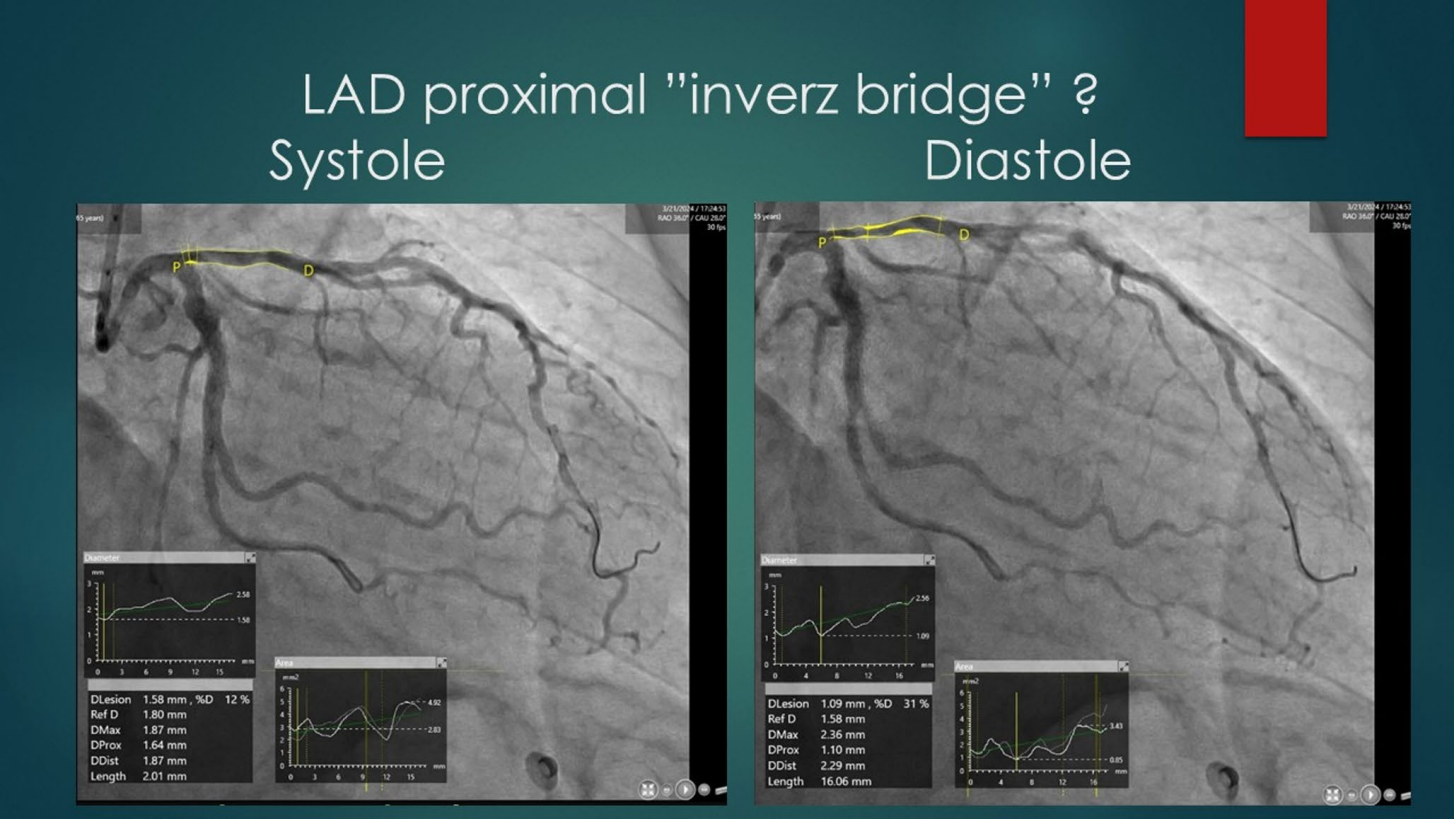
Quantitative coronary angiography of the systolisc and diastolic frames.

At the end of the intervention, an iatrogenic dissection of the left main extending into the LAD was identified, likely due to deep engagement of the guiding catheter (Fig. 1, Supplementary Video 1). IVUS imaging confirmed a Type 2 dissection with an intimal flap extending from the LM into the proximal LAD (Supplementary Video 2).

**Fig. 2:**
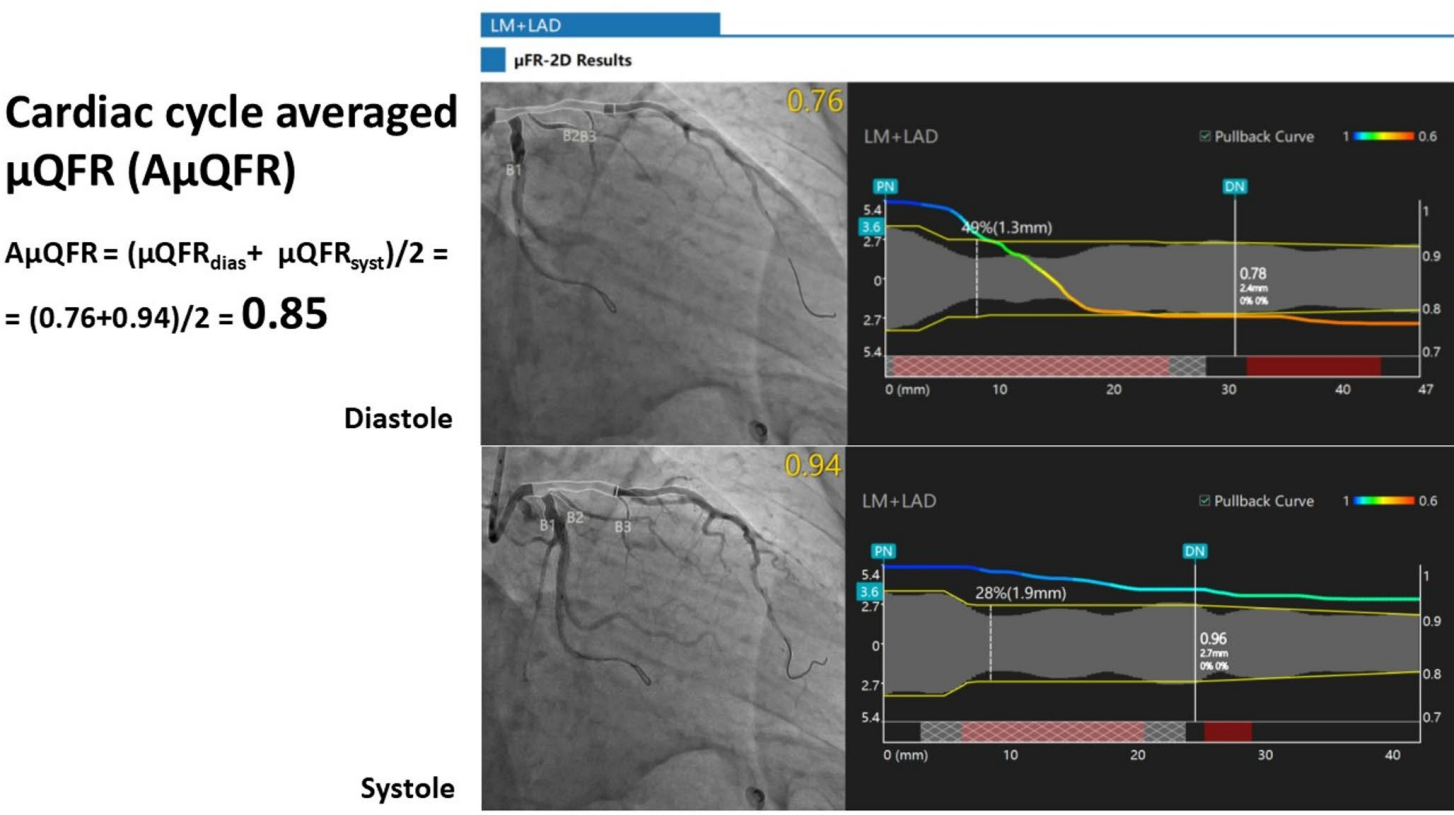
Calculation of the cardiac cycle avaraged QFR.

Angiographic assessment revealed an unusual dynamic pattern with more pronounced luminal narrowing during diastole. To evaluate functional significance, angiography-derived Murray law–based quantitative flow ratio (µQFR) was calculated separately for diastole and systole. The values were 0.76 and 0.94, respectively, resulting in a cardiac cycle-averaged µQFR (AµQFR) of 0.85 (Fig. 2).

**Fig. 3:**
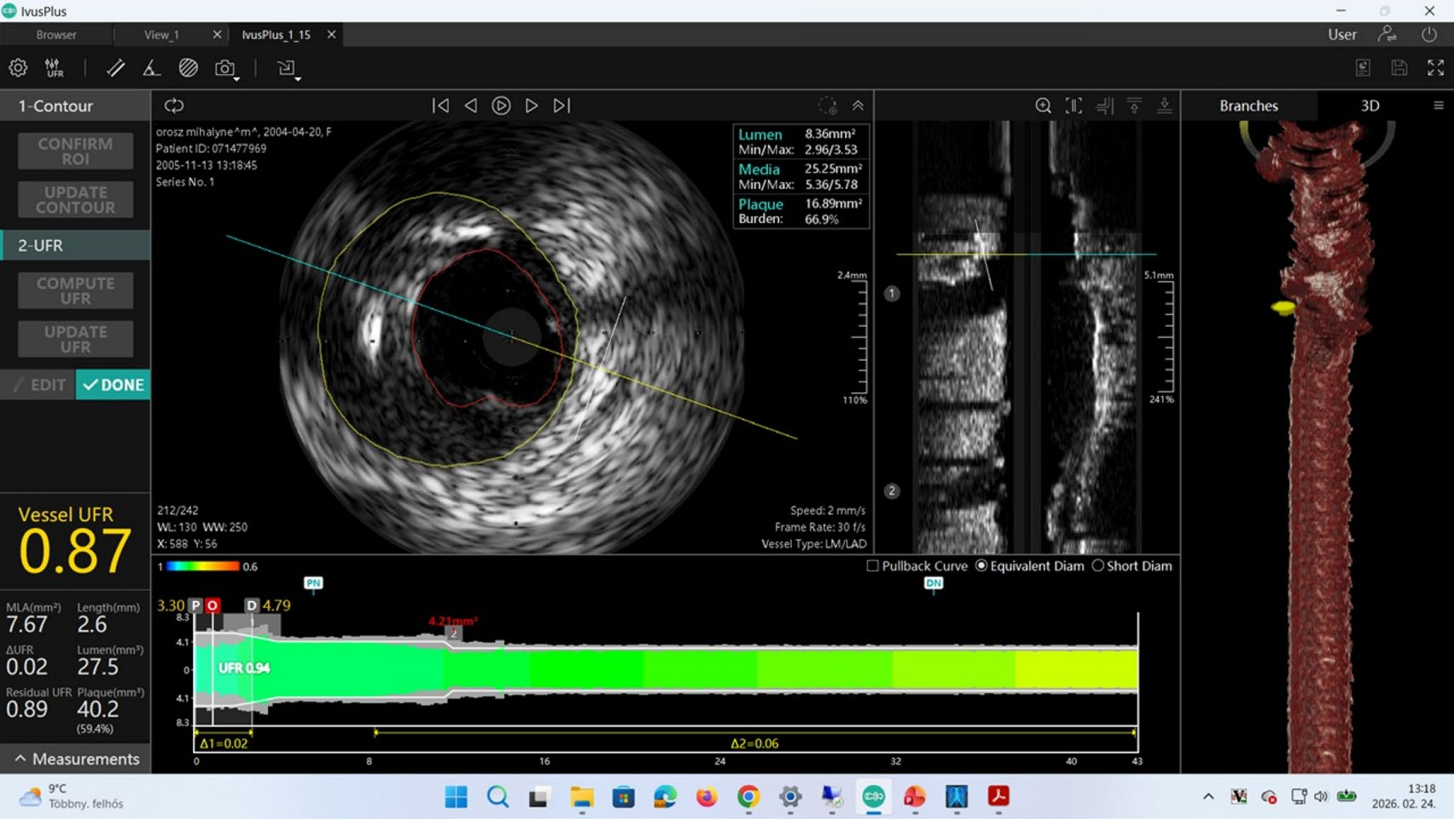
Ultrasonic flow ratio (UFR).

Additionally, IVUS-derived Ultrasonic Flow Ratio (UFR) was calculated, yielding a value of 0.87, confirming preserved coronary flow [4, 5] (Fig. 3).

Given the patient’s stability, absence of ischemic symptoms, and lack of significant physiological impairment, conservative management was chosen. The patient received dual antiplatelet therapy, statins, and guideline-directed heart failure therapy.

At 3-month follow-up, repeat angiography demonstrated complete healing of the dissection and resolution of dynamic luminal narrowing.

## Discussion

This case describes a novel presentation of iatrogenic LM dissection with predominantly diastolic coronary narrowing. While dynamic coronary stenosis is well described in myocardial bridging, it is typically systolic in nature. The inverse phenomenon observed in this case appears to be related to the behavior of the intimal flap.

We hypothesize that during systole, increased intracoronary pressure compresses the intimal flap against the vessel wall, reducing luminal obstruction. In contrast, during diastole, lower pressure allows the flap to protrude into the lumen, causing more significant narrowing.

This case underscores the importance of multimodality imaging. IVUS was essential for identifying the mechanism and extent of dissection. Angiography-derived µQFR allowed phase-specific physiological assessment, revealing significant variation between systole and diastole. The use of cardiac cycle-averaged µQFR provided a more comprehensive evaluation of flow limitation [2, 3].

Furthermore, IVUS-derived UFR offered an independent physiological assessment, corroborating the absence of hemodynamically significant impairment [4, 5].

Although LM dissections are often treated with bailout stenting, this case demonstrates that conservative management may be appropriate in selected patients with preserved coronary flow and clinical stability.

## Conclusion

Predominantly diastolic coronary narrowing due to iatrogenic LM dissection represents a previously unreported phenomenon. Multimodality imaging, including IVUS, µQFR, and UFR, enables accurate anatomical and functional assessment. In selected cases with preserved flow, conservative management may be a safe alternative to immediate intervention.

## Supplementary Information

Below is the link to the electronic supplementary material.

Supplementary Material 1

Supplementary Material 2

## Data Availability

No datasets were generated or analysed during the current study.
